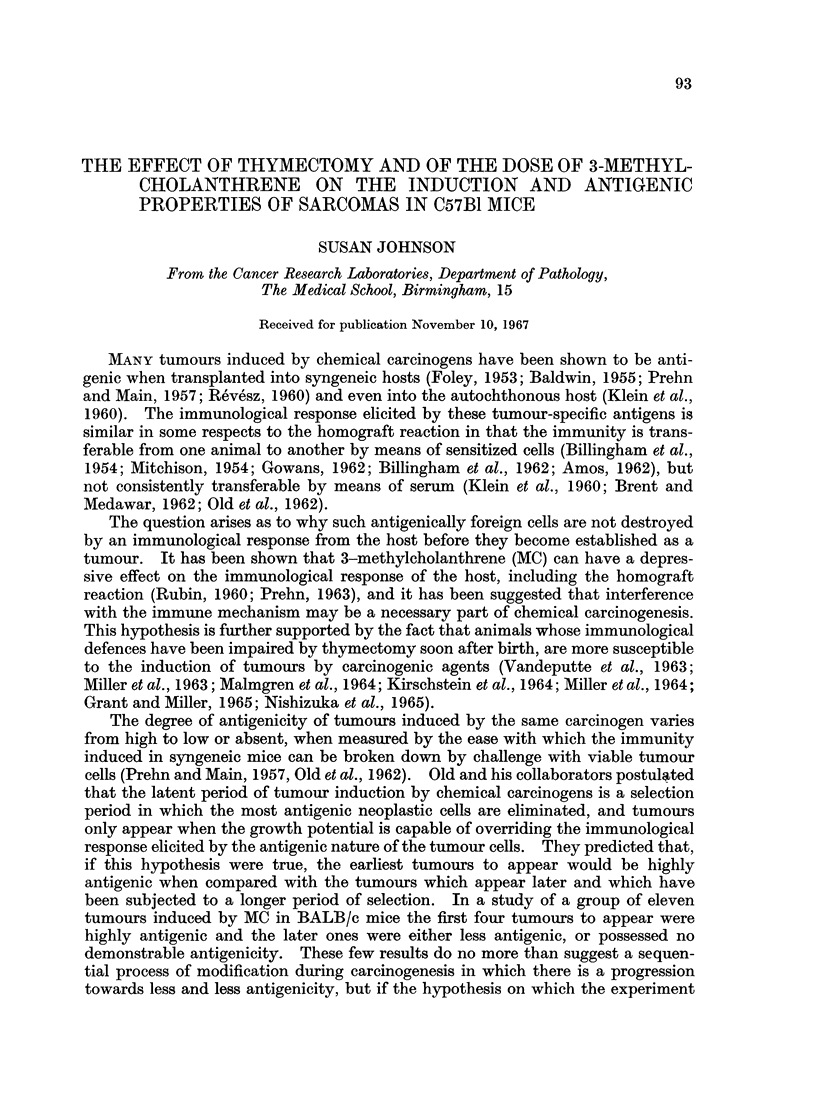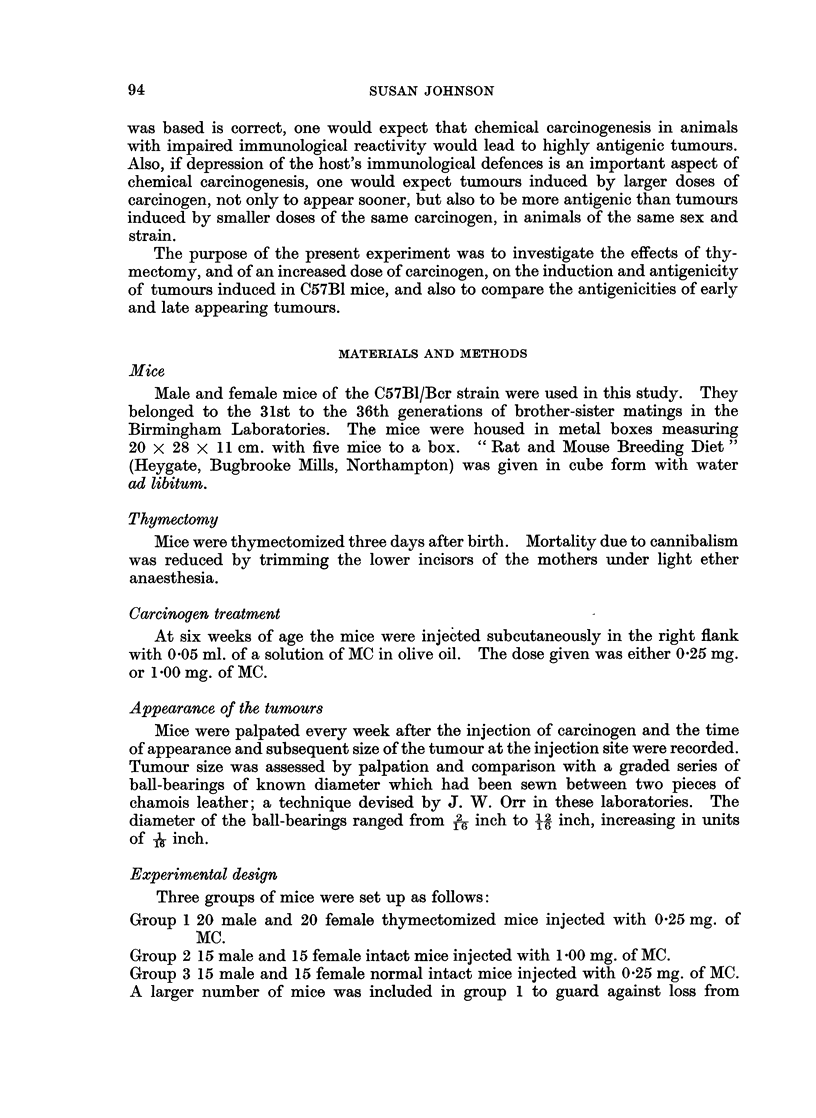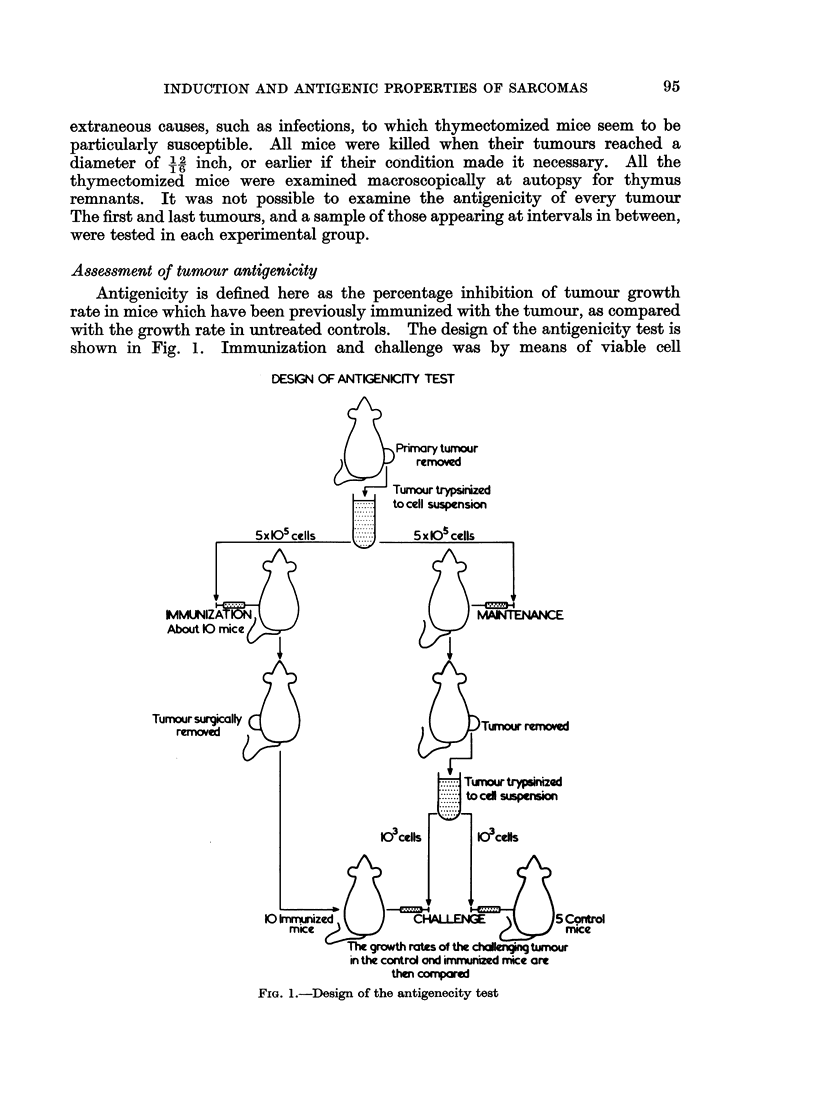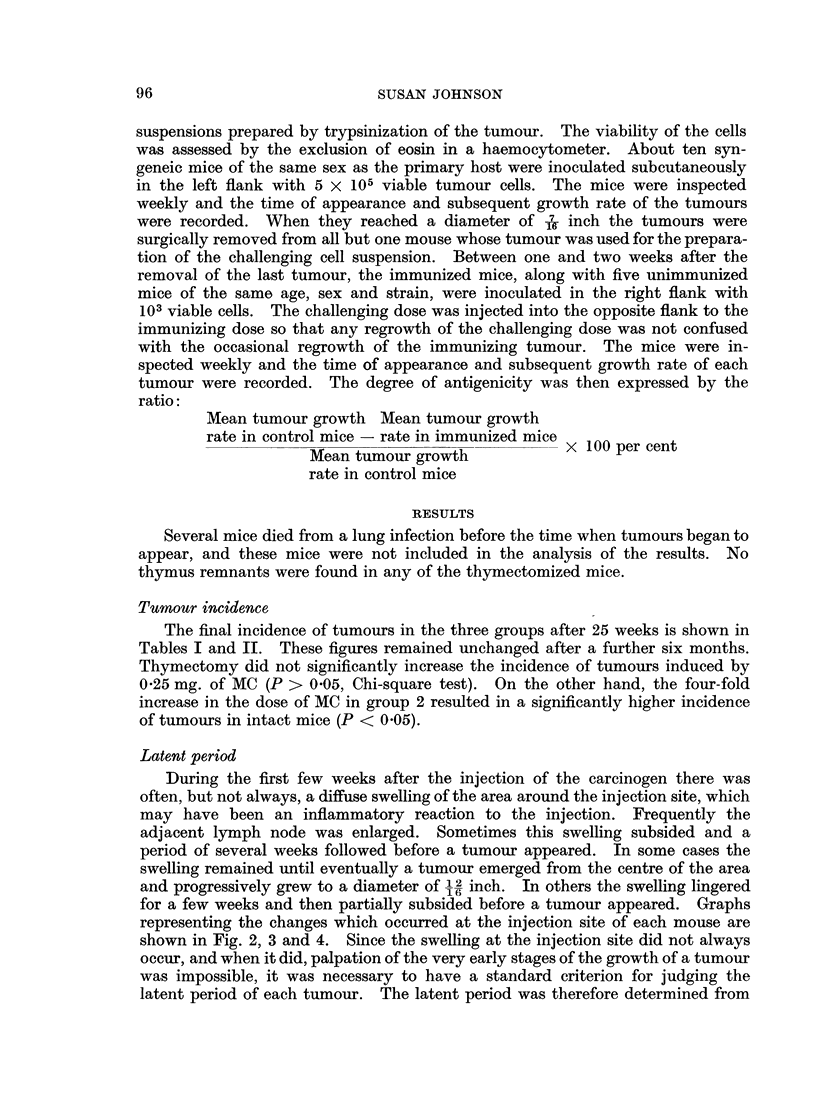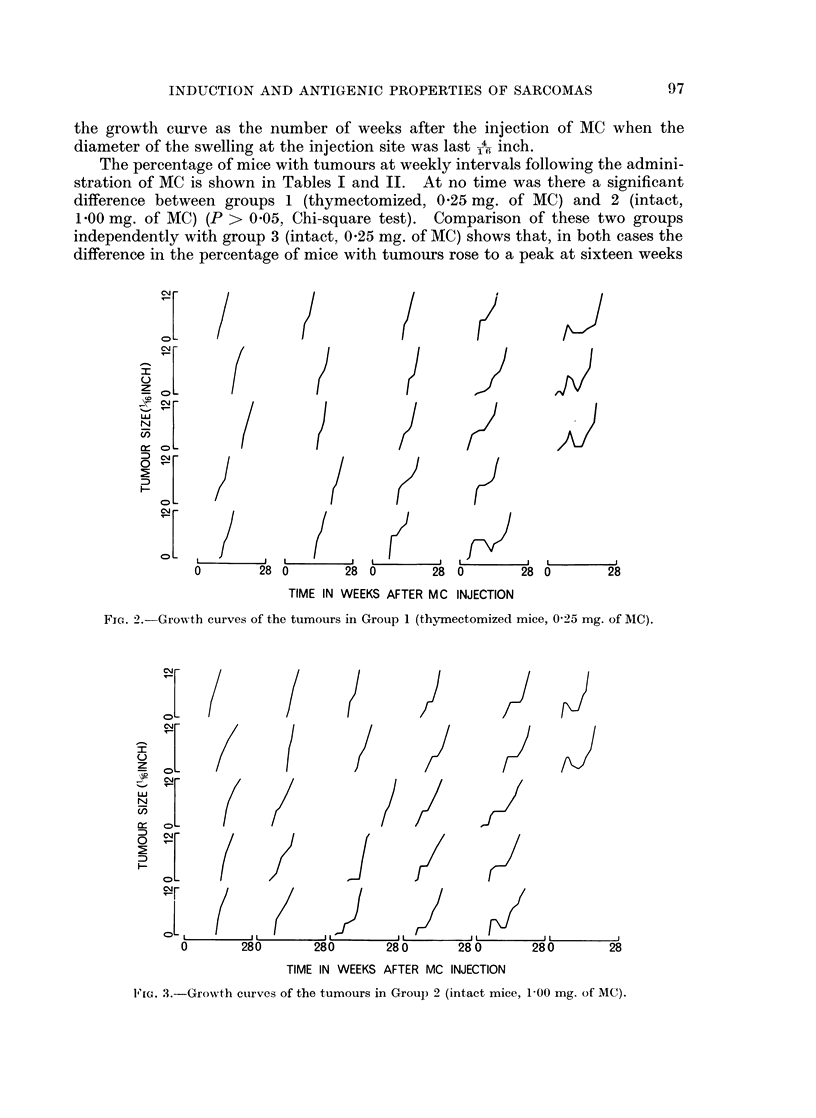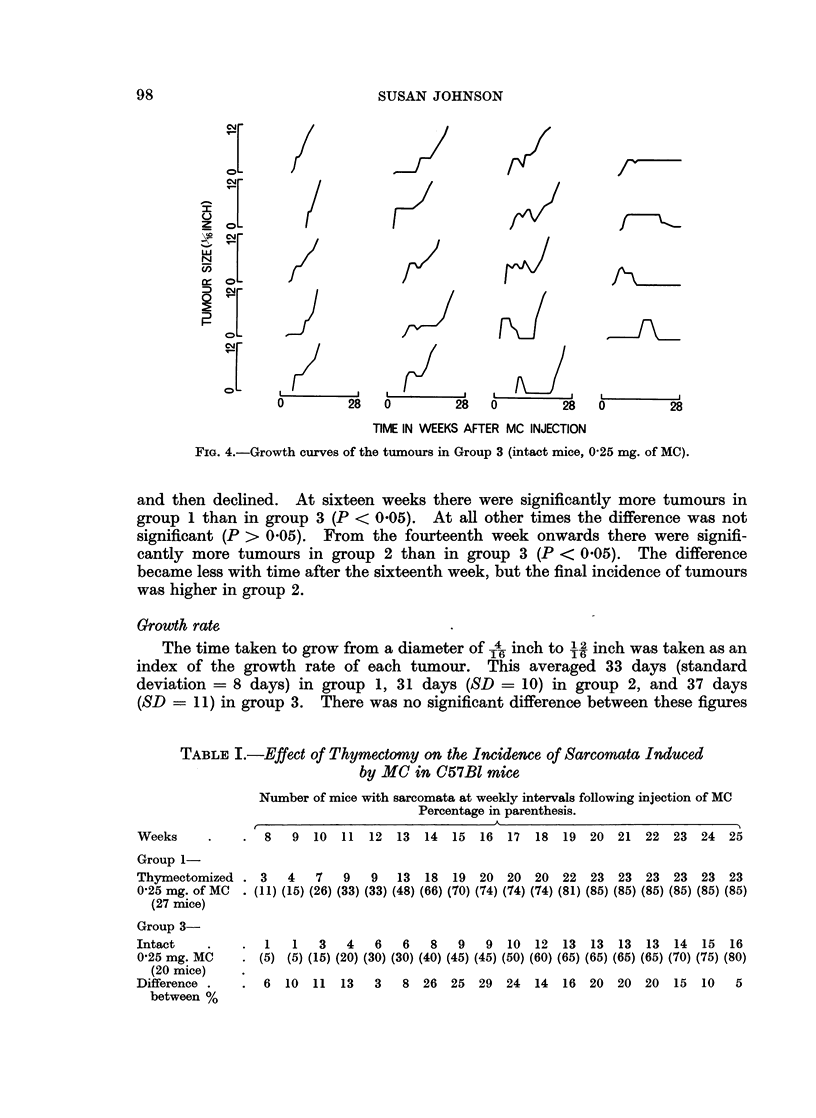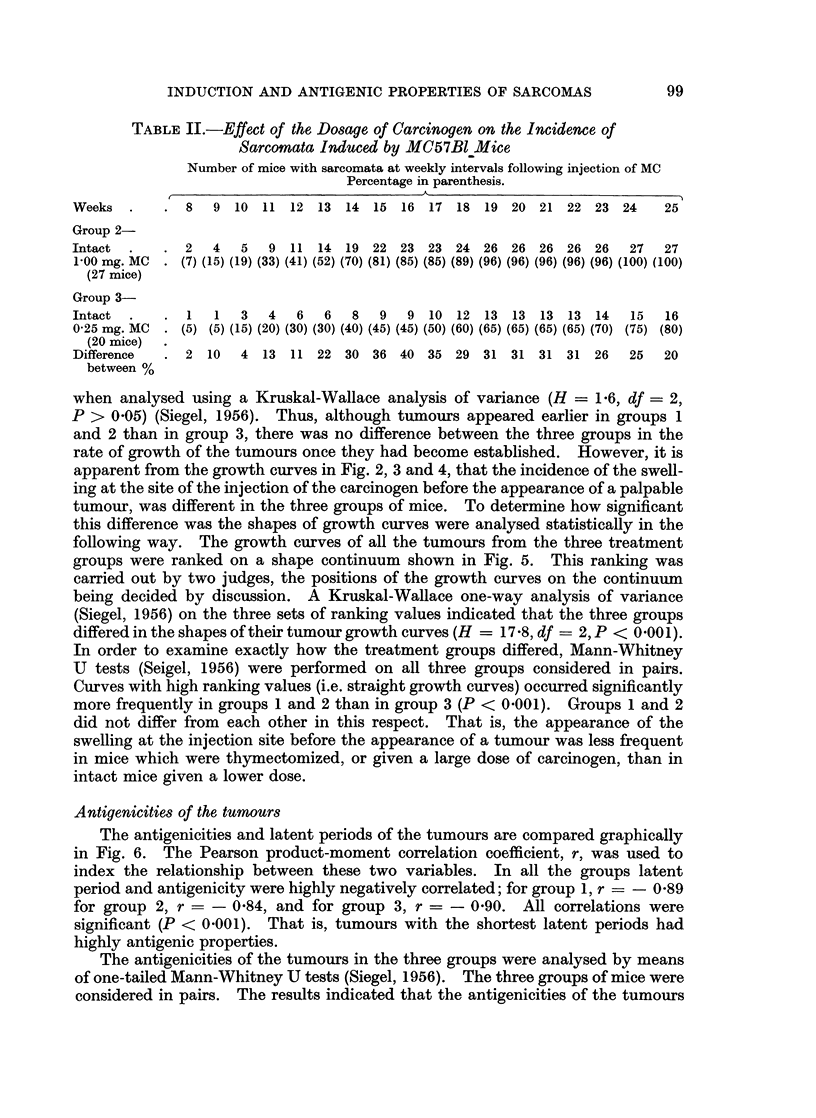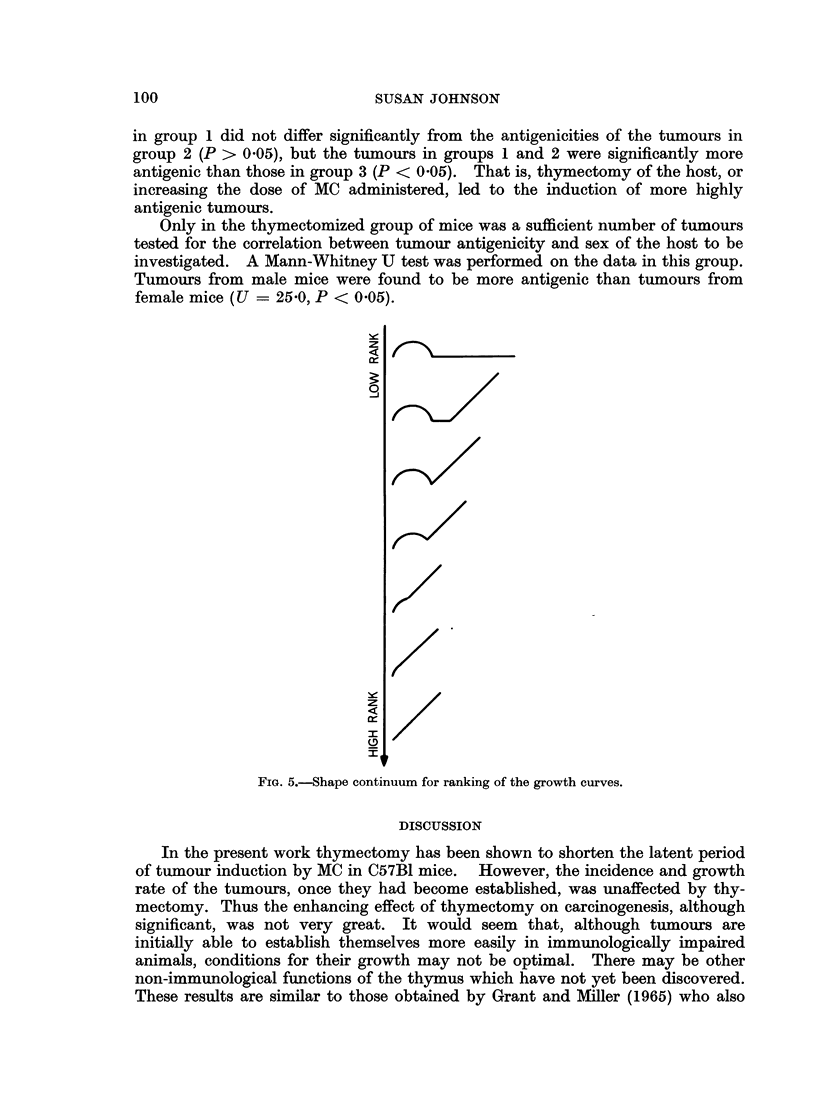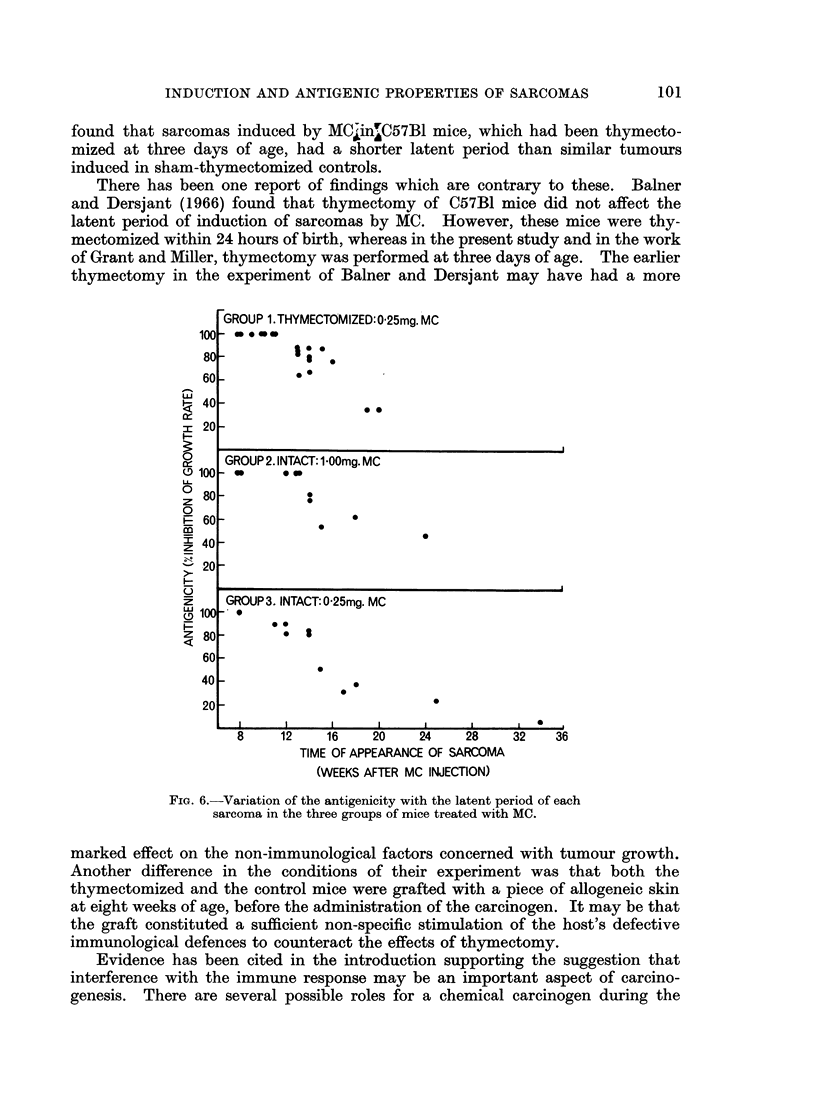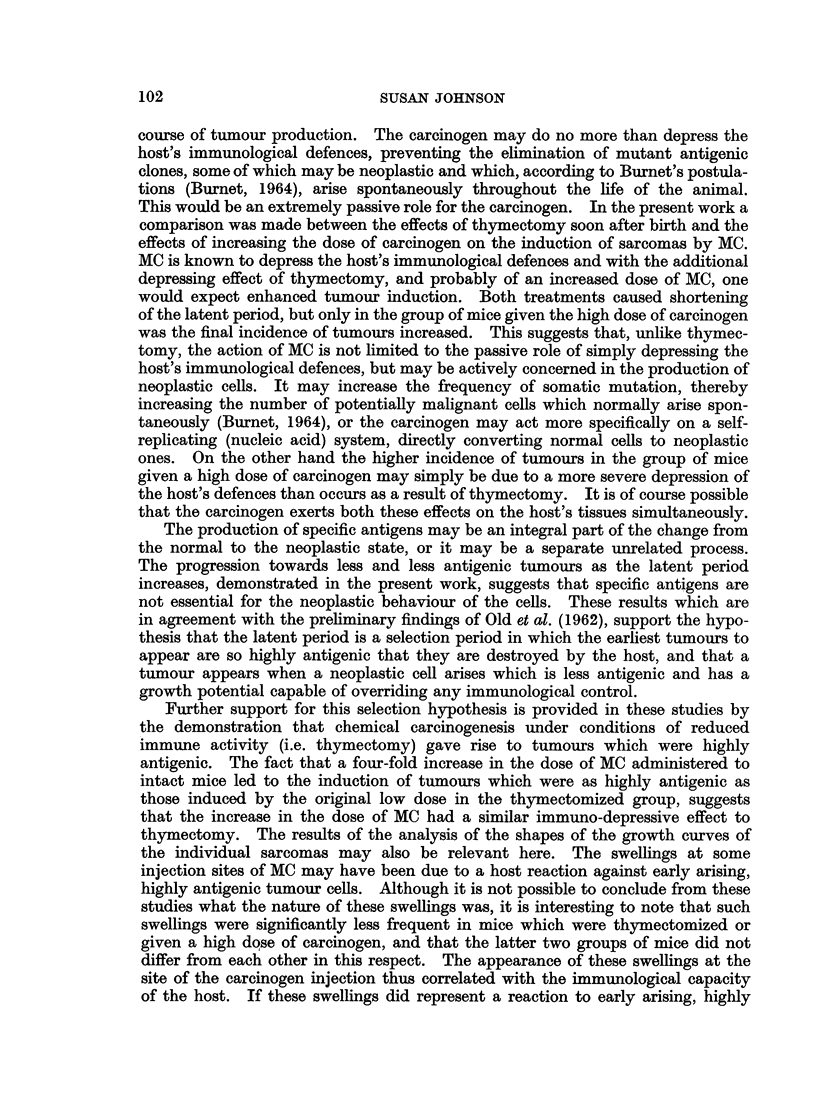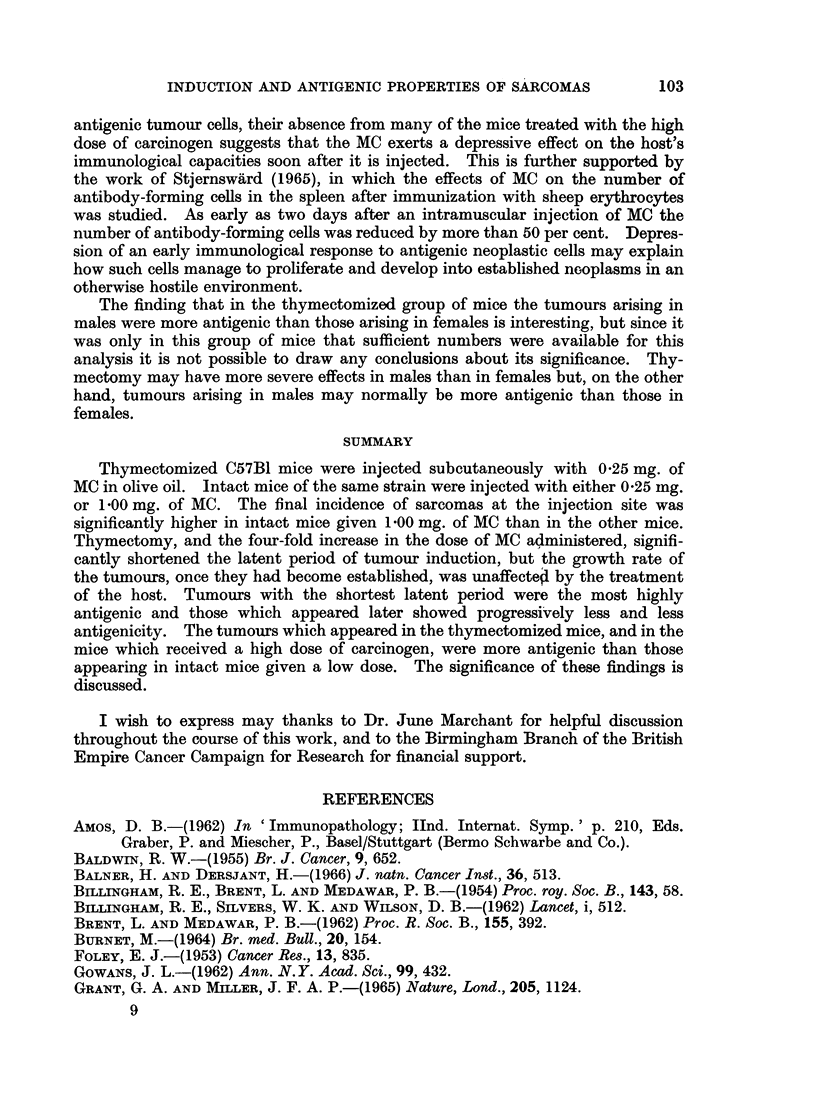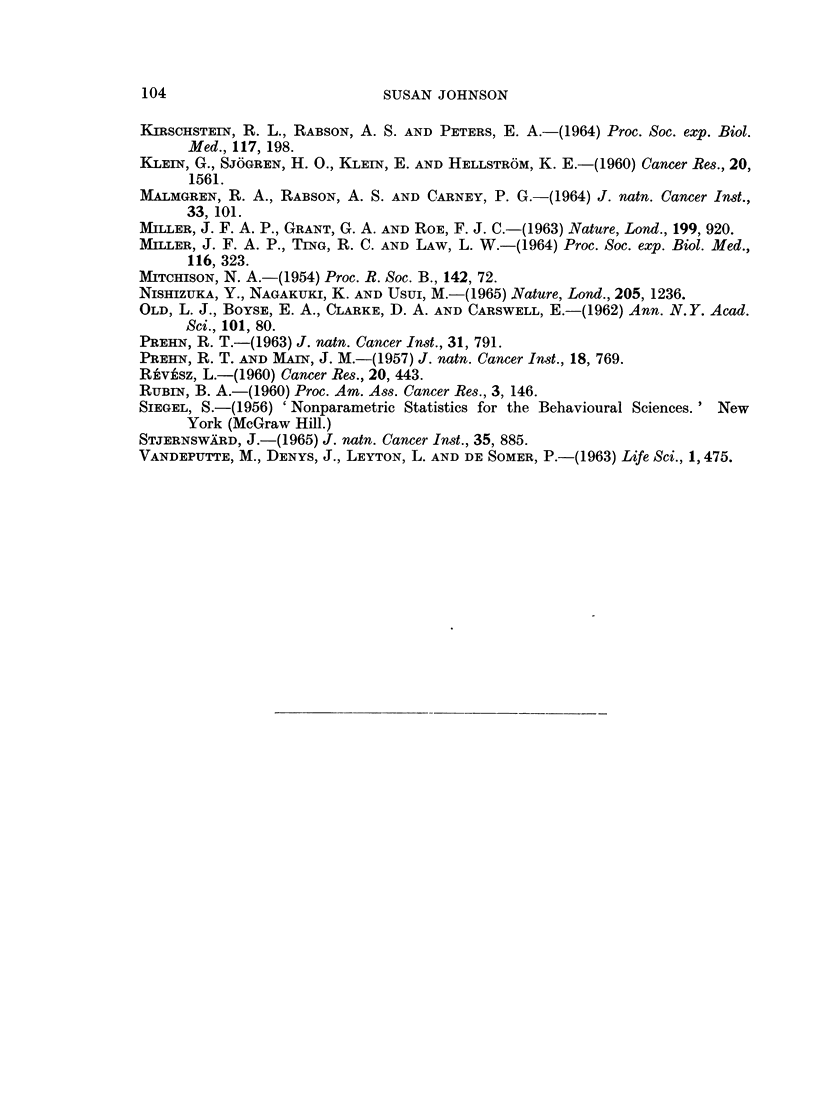# The effect of thymectomy and of the dose of 3-methylcholanthrene on the induction and antigenic properties of sarcomas in C56Bl mice.

**DOI:** 10.1038/bjc.1968.13

**Published:** 1968-03

**Authors:** S. Johnson


### THE EFFECT OF THYMECTOMY AND OF THE DOSE OF 3-METHYL-CHOLANTHRENE ON THE INDUCTION AND ANTIGENIC PROPERTIES OF SARCOMAS IN C57Bl MICE

SUSAN JOHNSON

*From the Cancer Research Laboratories, Department of Pathology, The Medical School, Birmingham,* 15

Received for publication November 10, 1967

MANY tumours induced by chemical carcinogens have been shown to be antigenic when transplanted into syngeneic hosts (Foley, 1953; Baldwin, 1955; Prehn and Main, 1957; Révész, 1960) and even into the autochthonous host (Klein *et al.*, 1960). The immunological response elicited by these tumour-specific antigens is similar in some respects to the homograft reaction in that the immunity is transferable from one animal to another by means of sensitized cells (Billingham *et al.*, 1954; Mitchison, 1954; Gowans, 1962; Billingham *et al.*, 1962; Amos, 1962), but not consistently transferable by means of serum (Klein *et al.*, 1960; Brent and Medawar, 1962; Old *et al.*, 1962).

The question arises as to why such antigenically foreign cells are not destroyed by an immunological response from the host before they become established as a tumour. It has been shown that 3–methylcholanthrene (MC) can have a depressive effect on the immunological response of the host, including the homograft reaction (Rubin, 1960; Prehn, 1963), and it has been suggested that interference with the immune mechanism may be a necessary part of chemical carcinogenesis. This hypothesis is further supported by the fact that animals whose immunological defences have been impaired by thymectomy soon after birth, are more susceptible to the induction of tumours by carcinogenic agents (Vandeputte *et al.*, 1963; Miller *et al.*, 1963; Malmgren *et al.*, 1964; Kirschstein *et al.*, 1964; Miller *et al.*, 1964; Grant and Miller, 1965; Nishizuka *et al.*, 1965).

The degree of antigenicity of tumours induced by the same carcinogen varies from high to low or absent, when measured by the ease with which the immunity induced in syngeneic mice can be broken down by challenge with viable tumour cells (Prehn and Main, 1957, Old *et al.*, 1962). Old and his collaborators postulated that the latent period of tumour induction by chemical carcinogens is a selection period in which the most antigenic neoplastic cells are eliminated, and tumours only appear when the growth potential is capable of overriding the immunological response elicited by the antigenic nature of the tumour cells. They predicted that, if this hypothesis were true, the earliest tumours to appear would be highly antigenic when compared with the tumours which appear later and which have been subjected to a longer period of selection. In a study of a group of eleven tumours induced by MC in BALB/c mice the first four tumours to appear were highly antigenic and the later ones were either less antigenic, or possessed no demonstrable antigenicity. These few results do no more than suggest a sequential process of modification during carcinogenesis in which there is a progression towards less and less antigenicity, but if the hypothesis on which the experiment was based is correct, one would expect that chemical carcinogenesis in animals with impaired immunological reactivity would lead to highly antigenic tumours. Also, if depression of the host's immunological defences is an important aspect of chemical carcinogenesis, one would expect tumours induced by larger doses of carcinogen, not only to appear sooner, but also to be more antigenic than tumours induced by smaller doses of the same carcinogen, in animals of the same sex and strain.

The purpose of the present experiment was to investigate the effects of thymectomy, and of an increased dose of carcinogen, on the induction and antigenicity of tumours induced in C57Bl mice, and also to compare the antigenicities of early and late appearing tumours.

### MATERIALS AND METHODS

### *Mice*

Male and female mice of the C57Bl/Bcr strain were used in this study. They belonged to the 31st to the 36th generations of brother-sister matings in the Birmingham Laboratories. The mice were housed in metal boxes measuring 20 × 28 × 11 cm. with five mice to a box. " Rat and Mouse Breeding Diet " (Heygate, Bugbrooke Mills, Northampton) was given in cube form with water *ad libitum.*

### *Thymectomy*

Mice were thymectomized three days after birth. Mortality due to cannibalism was reduced by trimming the lower incisors of the mothers under light ether anaesthesia.

### *Carcinogen treatment*

At six weeks of age the mice were injected subcutaneously in the right flank with 0·05 ml. of a solution of MC in olive oil. The dose given was either 0·25 mg. or 1·00 mg. of MC.

### *Appearance of the tumours*

Mice were palpated every week after the injection of carcinogen and the time of appearance and subsequent size of the tumour at the injection site were recorded. Tumour size was assessed by palpation and comparison with a graded series of ball-bearings of known diameter which had been sewn between two pieces of chamois leather; a technique devised by J. W. Orr in these laboratories. The diameter of the ball-bearings ranged from $\frac{2}{16}$ inch to $\frac{12}{16}$ inch, increasing in units of $\frac{1}{16}$ inch.

### *Experimental design*

Three groups of mice were set up as follows:

Group 1 20 male and 20 female thymectomized mice injected with 0·25 mg. of MC.
Group 2 15 male and 15 female intact mice injected with 1·00 mg. of MC.
Group 3 15 male and 15 female normal intact mice injected with 0·25 mg. of MC.

A larger number of mice was included in group 1 to guard against loss from extraneous causes, such as infections, to which thymectomized mice seem to be particularly susceptible. All mice were killed when their tumours reached a diameter of $\frac{12}{16}$ inch, or earlier if their condition made it necessary. All the thymectomized mice were examined macroscopically at autopsy for thymus remnants. It was not possible to examine the antigenicity of every tumour The first and last tumours, and a sample of those appearing at intervals in between, were tested in each experimental group.

*Assessment of tumour antigenicity*

Antigenicity is defined here as the percentage inhibition of tumour growth rate in mice which have been previously immunized with the tumour, as compared with the growth rate in untreated controls. The design of the antigenicity test is shown in Fig. 1. Immunization and challenge was by means of viable cell

FIG. 1.—Design of the antigenecity test suspensions prepared by trypsinization of the tumour. The viability of the cells was assessed by the exclusion of eosin in a haemocytometer. About ten syngeneic mice of the same sex as the primary host were inoculated subcutaneously in the left flank with $5 \times 10^5$ viable tumour cells. The mice were inspected weekly and the time of appearance and subsequent growth rate of the tumours were recorded. When they reached a diameter of $\frac{7}{16}$ inch the tumours were surgically removed from all but one mouse whose tumour was used for the preparation of the challenging cell suspension. Between one and two weeks after the removal of the last tumour, the immunized mice, along with five unimmunized mice of the same age, sex and strain, were inoculated in the right flank with $10^3$ viable cells. The challenging dose was injected into the opposite flank to the immunizing dose so that any regrowth of the challenging dose was not confused with the occasional regrowth of the immunizing tumour. The mice were inspected weekly and the time of appearance and subsequent growth rate of each tumour were recorded. The degree of antigenicity was then expressed by the ratio:

$$\frac{\begin{array}{c}\text{Mean tumour growth} \\ \text{rate in control mice}\end{array} - \begin{array}{c}\text{Mean tumour growth} \\ \text{rate in immunized mice}\end{array}}{\begin{array}{c}\text{Mean tumour growth} \\ \text{rate in control mice}\end{array}} \times 100 \text{ per cent}$$

### RESULTS

Several mice died from a lung infection before the time when tumours began to appear, and these mice were not included in the analysis of the results. No thymus remnants were found in any of the thymectomized mice.

### *Tumour incidence*

The final incidence of tumours in the three groups after 25 weeks is shown in Tables I and II. These figures remained unchanged after a further six months. Thymectomy did not significantly increase the incidence of tumours induced by 0·25 mg. of MC ($P > 0{\cdot}05$, Chi-square test). On the other hand, the four-fold increase in the dose of MC in group 2 resulted in a significantly higher incidence of tumours in intact mice ($P < 0{\cdot}05$).

### *Latent period*

During the first few weeks after the injection of the carcinogen there was often, but not always, a diffuse swelling of the area around the injection site, which may have been an inflammatory reaction to the injection. Frequently the adjacent lymph node was enlarged. Sometimes this swelling subsided and a period of several weeks followed before a tumour appeared. In some cases the swelling remained until eventually a tumour emerged from the centre of the area and progressively grew to a diameter of $\frac{12}{16}$ inch. In others the swelling lingered for a few weeks and then partially subsided before a tumour appeared. Graphs representing the changes which occurred at the injection site of each mouse are shown in Fig. 2, 3 and 4. Since the swelling at the injection site did not always occur, and when it did, palpation of the very early stages of the growth of a tumour was impossible, it was necessary to have a standard criterion for judging the latent period of each tumour. The latent period was therefore determined from the growth curve as the number of weeks after the injection of MC when the diameter of the swelling at the injection site was last $\frac{4}{16}$ inch.

The percentage of mice with tumours at weekly intervals following the administration of MC is shown in Tables I and II. At no time was there a significant difference between groups 1 (thymectomized, 0·25 mg. of MC) and 2 (intact, 1·00 mg. of MC) ($P > 0{\cdot}05$, Chi-square test). Comparison of these two groups independently with group 3 (intact, 0·25 mg. of MC) shows that, in both cases the difference in the percentage of mice with tumours rose to a peak at sixteen weeks

FIG. 2.—Growth curves of the tumours in Group 1 (thymectomized mice, 0·25 mg. of MC).

FIG. 3.—Growth curves of the tumours in Group 2 (intact mice, 1·00 mg. of MC).

FIG. 4.—Growth curves of the tumours in Group 3 (intact mice, 0·25 mg. of MC).

and then declined. At sixteen weeks there were significantly more tumours in group 1 than in group 3 ($P < 0{\cdot}05$). At all other times the difference was not significant ($P > 0{\cdot}05$). From the fourteenth week onwards there were significantly more tumours in group 2 than in group 3 ($P < 0{\cdot}05$). The difference became less with time after the sixteenth week, but the final incidence of tumours was higher in group 2.

*Growth rate*

The time taken to grow from a diameter of $\frac{4}{16}$ inch to $\frac{12}{16}$ inch was taken as an index of the growth rate of each tumour. This averaged 33 days (standard deviation = 8 days) in group 1, 31 days ($SD = 10$) in group 2, and 37 days ($SD = 11$) in group 3. There was no significant difference between these figures

TABLE I.—*Effect of Thymectomy on the Incidence of Sarcomata Induced by MC in C57Bl mice*

| | Number of mice with sarcomata at weekly intervals following injection of MC Percentage in parenthesis. | | | | | | | | | | | | | | | | | |
|---|---|---|---|---|---|---|---|---|---|---|---|---|---|---|---|---|---|---|
| Weeks | 8 | 9 | 10 | 11 | 12 | 13 | 14 | 15 | 16 | 17 | 18 | 19 | 20 | 21 | 22 | 23 | 24 | 25 |
| Group 1— | | | | | | | | | | | | | | | | | | |
| Thymectomized | 3 | 4 | 7 | 9 | 9 | 13 | 18 | 19 | 20 | 20 | 20 | 22 | 23 | 23 | 23 | 23 | 23 | 23 |
| 0·25 mg. of MC (27 mice) | (11) | (15) | (26) | (33) | (33) | (48) | (66) | (70) | (74) | (74) | (74) | (81) | (85) | (85) | (85) | (85) | (85) | (85) |
| Group 3— | | | | | | | | | | | | | | | | | | |
| Intact | 1 | 1 | 3 | 4 | 6 | 6 | 8 | 9 | 9 | 10 | 12 | 13 | 13 | 13 | 13 | 14 | 15 | 16 |
| 0·25 mg. MC (20 mice) | (5) | (5) | (15) | (20) | (30) | (30) | (40) | (45) | (45) | (50) | (60) | (65) | (65) | (65) | (65) | (70) | (75) | (80) |
| Difference between % | 6 | 10 | 11 | 13 | 3 | 8 | 26 | 25 | 29 | 24 | 14 | 16 | 20 | 20 | 20 | 15 | 10 | 5 |

TABLE II.—*Effect of the Dosage of Carcinogen on the Incidence of Sarcomata Induced by MC57Bl Mice*

Number of mice with sarcomata at weekly intervals following injection of MC
Percentage in parenthesis.

| Weeks | 8 | 9 | 10 | 11 | 12 | 13 | 14 | 15 | 16 | 17 | 18 | 19 | 20 | 21 | 22 | 23 | 24 | 25 |
|---|---|---|---|---|---|---|---|---|---|---|---|---|---|---|---|---|---|---|
| Group 2— | | | | | | | | | | | | | | | | | | |
| Intact | 2 | 4 | 5 | 9 | 11 | 14 | 19 | 22 | 23 | 23 | 24 | 26 | 26 | 26 | 26 | 26 | 27 | 27 |
| 1·00 mg. MC (27 mice) | (7) | (15) | (19) | (33) | (41) | (52) | (70) | (81) | (85) | (85) | (89) | (96) | (96) | (96) | (96) | (96) | (100) | (100) |
| Group 3— | | | | | | | | | | | | | | | | | | |
| Intact | 1 | 1 | 3 | 4 | 6 | 6 | 8 | 9 | 9 | 10 | 12 | 13 | 13 | 13 | 13 | 14 | 15 | 16 |
| 0·25 mg. MC (20 mice) | (5) | (5) | (15) | (20) | (30) | (30) | (40) | (45) | (45) | (50) | (60) | (65) | (65) | (65) | (65) | (70) | (75) | (80) |
| Difference between % | 2 | 10 | 4 | 13 | 11 | 22 | 30 | 36 | 40 | 35 | 29 | 31 | 31 | 31 | 31 | 26 | 25 | 20 |

when analysed using a Kruskal-Wallace analysis of variance ($H = 1 \cdot 6$, $df = 2$, $P > 0 \cdot 05$) (Siegel, 1956). Thus, although tumours appeared earlier in groups 1 and 2 than in group 3, there was no difference between the three groups in the rate of growth of the tumours once they had become established. However, it is apparent from the growth curves in Fig. 2, 3 and 4, that the incidence of the swelling at the site of the injection of the carcinogen before the appearance of a palpable tumour, was different in the three groups of mice. To determine how significant this difference was the shapes of growth curves were analysed statistically in the following way. The growth curves of all the tumours from the three treatment groups were ranked on a shape continuum shown in Fig. 5. This ranking was carried out by two judges, the positions of the growth curves on the continuum being decided by discussion. A Kruskal-Wallace one-way analysis of variance (Siegel, 1956) on the three sets of ranking values indicated that the three groups differed in the shapes of their tumour growth curves ($H = 17 \cdot 8$, $df = 2, P < 0 \cdot 001$). In order to examine exactly how the treatment groups differed, Mann-Whitney U tests (Seigel, 1956) were performed on all three groups considered in pairs. Curves with high ranking values (i.e. straight growth curves) occurred significantly more frequently in groups 1 and 2 than in group 3 ($P < 0 \cdot 001$). Groups 1 and 2 did not differ from each other in this respect. That is, the appearance of the swelling at the injection site before the appearance of a tumour was less frequent in mice which were thymectomized, or given a large dose of carcinogen, than in intact mice given a lower dose.

*Antigenicities of the tumours*

The antigenicities and latent periods of the tumours are compared graphically in Fig. 6. The Pearson product-moment correlation coefficient, $r$, was used to index the relationship between these two variables. In all the groups latent period and antigenicity were highly negatively correlated; for group 1, $r = - 0 \cdot 89$ for group 2, $r = - 0 \cdot 84$, and for group 3, $r = - 0 \cdot 90$. All correlations were significant ($P < 0 \cdot 001$). That is, tumours with the shortest latent periods had highly antigenic properties.

The antigenicities of the tumours in the three groups were analysed by means of one-tailed Mann-Whitney U tests (Siegel, 1956). The three groups of mice were considered in pairs. The results indicated that the antigenicities of the tumours in group 1 did not differ significantly from the antigenicities of the tumours in group 2 ($P > 0{\cdot}05$), but the tumours in groups 1 and 2 were significantly more antigenic than those in group 3 ($P < 0{\cdot}05$). That is, thymectomy of the host, or increasing the dose of MC administered, led to the induction of more highly antigenic tumours.

Only in the thymectomized group of mice was a sufficient number of tumours tested for the correlation between tumour antigenicity and sex of the host to be investigated. A Mann-Whitney U test was performed on the data in this group. Tumours from male mice were found to be more antigenic than tumours from female mice ($U = 25{\cdot}0$, $P < 0{\cdot}05$).

FIG. 5.—Shape continuum for ranking of the growth curves.

### DISCUSSION

In the present work thymectomy has been shown to shorten the latent period of tumour induction by MC in C57Bl mice. However, the incidence and growth rate of the tumours, once they had become established, was unaffected by thymectomy. Thus the enhancing effect of thymectomy on carcinogenesis, although significant, was not very great. It would seem that, although tumours are initially able to establish themselves more easily in immunologically impaired animals, conditions for their growth may not be optimal. There may be other non-immunological functions of the thymus which have not yet been discovered. These results are similar to those obtained by Grant and Miller (1965) who also found that sarcomas induced by MC in C57Bl mice, which had been thymectomized at three days of age, had a shorter latent period than similar tumours induced in sham-thymectomized controls.

There has been one report of findings which are contrary to these. Balner and Dersjant (1966) found that thymectomy of C57Bl mice did not affect the latent period of induction of sarcomas by MC. However, these mice were thymectomized within 24 hours of birth, whereas in the present study and in the work of Grant and Miller, thymectomy was performed at three days of age. The earlier thymectomy in the experiment of Balner and Dersjant may have had a more

Fig. 6.—Variation of the antigenicity with the latent period of each sarcoma in the three groups of mice treated with MC.

marked effect on the non-immunological factors concerned with tumour growth. Another difference in the conditions of their experiment was that both the thymectomized and the control mice were grafted with a piece of allogeneic skin at eight weeks of age, before the administration of the carcinogen. It may be that the graft constituted a sufficient non-specific stimulation of the host's defective immunological defences to counteract the effects of thymectomy.

Evidence has been cited in the introduction supporting the suggestion that interference with the immune response may be an important aspect of carcinogenesis. There are several possible roles for a chemical carcinogen during the course of tumour production. The carcinogen may do no more than depress the host's immunological defences, preventing the elimination of mutant antigenic clones, some of which may be neoplastic and which, according to Burnet's postulations (Burnet, 1964), arise spontaneously throughout the life of the animal. This would be an extremely passive role for the carcinogen. In the present work a comparison was made between the effects of thymectomy soon after birth and the effects of increasing the dose of carcinogen on the induction of sarcomas by MC. MC is known to depress the host's immunological defences and with the additional depressing effect of thymectomy, and probably of an increased dose of MC, one would expect enhanced tumour induction. Both treatments caused shortening of the latent period, but only in the group of mice given the high dose of carcinogen was the final incidence of tumours increased. This suggests that, unlike thymectomy, the action of MC is not limited to the passive role of simply depressing the host's immunological defences, but may be actively concerned in the production of neoplastic cells. It may increase the frequency of somatic mutation, thereby increasing the number of potentially malignant cells which normally arise spontaneously (Burnet, 1964), or the carcinogen may act more specifically on a self-replicating (nucleic acid) system, directly converting normal cells to neoplastic ones. On the other hand the higher incidence of tumours in the group of mice given a high dose of carcinogen may simply be due to a more severe depression of the host's defences than occurs as a result of thymectomy. It is of course possible that the carcinogen exerts both these effects on the host's tissues simultaneously.

The production of specific antigens may be an integral part of the change from the normal to the neoplastic state, or it may be a separate unrelated process. The progression towards less and less antigenic tumours as the latent period increases, demonstrated in the present work, suggests that specific antigens are not essential for the neoplastic behaviour of the cells. These results which are in agreement with the preliminary findings of Old *et al.* (1962), support the hypothesis that the latent period is a selection period in which the earliest tumours to appear are so highly antigenic that they are destroyed by the host, and that a tumour appears when a neoplastic cell arises which is less antigenic and has a growth potential capable of overriding any immunological control.

Further support for this selection hypothesis is provided in these studies by the demonstration that chemical carcinogenesis under conditions of reduced immune activity (i.e. thymectomy) gave rise to tumours which were highly antigenic. The fact that a four-fold increase in the dose of MC administered to intact mice led to the induction of tumours which were as highly antigenic as those induced by the original low dose in the thymectomized group, suggests that the increase in the dose of MC had a similar immuno-depressive effect to thymectomy. The results of the analysis of the shapes of the growth curves of the individual sarcomas may also be relevant here. The swellings at some injection sites of MC may have been due to a host reaction against early arising, highly antigenic tumour cells. Although it is not possible to conclude from these studies what the nature of these swellings was, it is interesting to note that such swellings were significantly less frequent in mice which were thymectomized or given a high dose of carcinogen, and that the latter two groups of mice did not differ from each other in this respect. The appearance of these swellings at the site of the carcinogen injection thus correlated with the immunological capacity of the host. If these swellings did represent a reaction to early arising, highly antigenic tumour cells, their absence from many of the mice treated with the high dose of carcinogen suggests that the MC exerts a depressive effect on the host's immunological capacities soon after it is injected. This is further supported by the work of Stjernswärd (1965), in which the effects of MC on the number of antibody-forming cells in the spleen after immunization with sheep erythrocytes was studied. As early as two days after an intramuscular injection of MC the number of antibody-forming cells was reduced by more than 50 per cent. Depression of an early immunological response to antigenic neoplastic cells may explain how such cells manage to proliferate and develop into established neoplasms in an otherwise hostile environment.

The finding that in the thymectomized group of mice the tumours arising in males were more antigenic than those arising in females is interesting, but since it was only in this group of mice that sufficient numbers were available for this analysis it is not possible to draw any conclusions about its significance. Thymectomy may have more severe effects in males than in females but, on the other hand, tumours arising in males may normally be more antigenic than those in females.

SUMMARY

Thymectomized C57Bl mice were injected subcutaneously with 0·25 mg. of MC in olive oil. Intact mice of the same strain were injected with either 0·25 mg. or 1·00 mg. of MC. The final incidence of sarcomas at the injection site was significantly higher in intact mice given 1·00 mg. of MC than in the other mice. Thymectomy, and the four-fold increase in the dose of MC administered, significantly shortened the latent period of tumour induction, but the growth rate of the tumours, once they had become established, was unaffected by the treatment of the host. Tumours with the shortest latent period were the most highly antigenic and those which appeared later showed progressively less and less antigenicity. The tumours which appeared in the thymectomized mice, and in the mice which received a high dose of carcinogen, were more antigenic than those appearing in intact mice given a low dose. The significance of these findings is discussed.

I wish to express may thanks to Dr. June Marchant for helpful discussion throughout the course of this work, and to the Birmingham Branch of the British Empire Cancer Campaign for Research for financial support.

### REFERENCES

AMOS, D. B.—(1962) *In* ' Immunopathology; IInd. Internat. Symp. ' p. 210, Eds. Graber, P. and Miescher, P., Basel/Stuttgart (Bermo Schwarbe and Co.).
BALDWIN, R. W.—(1955) *Br. J. Cancer*, **9**, 652.
BALNER, H. AND DERSJANT, H.—(1966) *J. natn. Cancer Inst.*, **36**, 513.
BILLINGHAM, R. E., BRENT, L. AND MEDAWAR, P. B.—(1954) *Proc. roy. Soc. B.*, **143**, 58.
BILLINGHAM, R. E., SILVERS, W. K. AND WILSON, D. B.—(1962) *Lancet*, i, 512.
BRENT, L. AND MEDAWAR, P. B.—(1962) *Proc. R. Soc.* B., **155**, 392.
BURNET, M.—(1964) *Br. med. Bull.*, **20**, 154.
FOLEY, E. J.—(1953) *Cancer Res.*, **13**, 835.
GOWANS, J. L.—(1962) *Ann. N.Y. Acad. Sci.*, **99**, 432.
GRANT, G. A. AND MILLER, J. F. A. P.—(1965) *Nature, Lond.*, **205**, 1124.

Kirschstein, R. L., Rabson, A. S. and Peters, E. A.—(1964) *Proc. Soc. exp. Biol. Med.*, **117**, 198.
Klein, G., Sjögren, H. O., Klein, E. and Hellström, K. E.—(1960) *Cancer Res.*, **20**, 1561.
Malmgren, R. A., Rabson, A. S. and Carney, P. G.—(1964) *J. natn. Cancer Inst.*, **33**, 101.
Miller, J. F. A. P., Grant, G. A. and Roe, F. J. C.—(1963) *Nature, Lond.*, **199**, 920.
Miller, J. F. A. P., Ting, R. C. and Law, L. W.—(1964) *Proc. Soc. exp. Biol. Med.*, **116**, 323.
Mitchison, N. A.—(1954) *Proc. R. Soc.* B., **142**, 72.
Nishizuka, Y., Nagakuki, K. and Usui, M.—(1965) *Nature, Lond.*, **205**, 1236.
Old, L. J., Boyse, E. A., Clarke, D. A. and Carswell, E.—(1962) *Ann. N.Y. Acad. Sci.*, **101**, 80.
Prehn, R. T.—(1963) *J. natn. Cancer Inst.*, **31**, 791.
Prehn, R. T. and Main, J. M.—(1957) *J. natn. Cancer Inst.*, **18**, 769.
Révész, L.—(1960) *Cancer Res.*, **20**, 443.
Rubin, B. A.—(1960) *Proc. Am. Ass. Cancer Res.*, **3**, 146.
Siegel, S.—(1956) ‘Nonparametric Statistics for the Behavioural Sciences.’ New York (McGraw Hill.)
Stjernswärd, J.—(1965) *J. natn. Cancer Inst.*, **35**, 885.
Vandeputte, M., Denys, J., Leyton, L. and de Somer, P.—(1963) *Life Sci.*, **1**, 475.